# Arabidopsis phenotyping reveals the importance of alcohol dehydrogenase and pyruvate decarboxylase for aerobic plant growth

**DOI:** 10.1038/s41598-020-73704-x

**Published:** 2020-10-07

**Authors:** Irene Ventura, Luca Brunello, Sergio Iacopino, Maria Cristina Valeri, Giacomo Novi, Tino Dornbusch, Pierdomenico Perata, Elena Loreti

**Affiliations:** 1 PlantLab, Institute of Life Sciences, Scuola Superiore Sant’Anna, Via Guidiccioni 10, 56010 San Giuliano Terme, Pisa Italy; 2 Institute of Agricultural Biology and Biotechnology, CNR, National Research Council, Via Moruzzi, 56124 Pisa, Italy; 3 LemnaTec GmbH, Nerscheider Weg 170, 52076 Aachen, Germany

**Keywords:** Plant physiology, Plant stress responses

## Abstract

Alcohol dehydrogenase (ADH) and pyruvate decarboxylase (PDC) are key to the establishment of the fermentative metabolism in plants during oxygen shortage. Most of the evidence that both ADH and PDC are required for plant tolerance to hypoxia comes from experiments performed by limiting oxygen in the environment, such as by exposing plants to gaseous hypoxia or to waterlogging or submergence. However, recent experiments have shown that hypoxic niches might exist in plants grown in aerobic conditions. Here, we investigated the importance of ADH and PDC for plant growth and development under aerobic conditions, long-term waterlogging and short-term submergence. Data were collected after optimizing the software associated with a commercially-available phenotyping instrument, to circumvent problems in separation of plants and background pixels based on colour features, which is not applicable for low-oxygen stressed plants due to the low colour contrast of leaves with the brownish soil. The results showed that the growth penalty associated with the lack of functional ADH1 or both PDC1 and PDC2 is greater under aerobic conditions than in hypoxia, highlighting the importance of fermentative metabolism in plants grown under normal, aerobic conditions.

## Introduction

Plant metabolism under low oxygen conditions, which is often due to soil waterlogging or submergence, relies to a large extent on the activation of fermentation, allowing ATP production when mitochondrial respiration fails. Fermentative metabolism recycles NAD^+^ from NADH produced during glycolysis, thus allowing ATP production through this pathway, including when the recycling of NAD through the electron transport chain in the mitochondria is hampered by limiting oxygen availability^1^. Although lactate is often produced at the onset of hypoxic conditions, through the activity of lactate dehydrogenase, alcoholic fermentation is predominant in most plant species^2^.

Alcoholic fermentation proceeds through the decarboxylation of pyruvate to acetaldehyde, which is followed by a reduction of acetaldehyde to ethanol. Two enzymes are required, namely pyruvate decarboxylase (PDC) and alcohol dehydrogenase (ADH). Alcohol dehydrogenase was one of the first plant genes to be studied at the molecular level. Its induction in plants subjected to anaerobic conditions dates back to the 1950s^3^ and it was soon discovered that maize ADH is among the few polypeptides that are synthesized under hypoxia^4^. These anaerobic polypeptides (ANPs) are simultaneously induced and condition the plant's ability to survive temporary flooding or waterlogging^5^. In 1969 it was demonstrated that a maize *adh* mutant cannot germinate under anaerobic conditions^6^, and this was soon confirmed in several other plant species.

The role of both ADH and PDC during low-oxygen stress is supported by genetic evidence^7,8^. A number of *adh* mutants have been identified in several plant species, including maize^9^ (*Zea mays*), tobacco^10^ (*Nicotiana plumbaginifolia*) and Arabidopsis^11^. In Arabidopsis, the *adh1* null mutant displays reduced hypoxia tolerance^8^ while ADH overexpression confers enhanced anoxia tolerance, also resulting in improved root growth^12^, but with no effect on Arabidopsis flooding survival^8^. In rice the elongation of the coleoptile under submergence is reduced in the *reduced adh activity* (*rad*) mutant. The *rad* mutant expresses a modified ADH1 protein as a consequence of a point mutation in the sequence of the gene, indicating that a fully functional ADH protein is important for submergence tolerance^13–15^. In maize, the *Adh1‐* mutant is very susceptible to anoxia^16–18^. All this experimental evidence has demonstrated that ADH has an essential role in fermentation and low‐oxygen tolerance.

The role of PDC has been investigated to a lesser extent. In Arabidopsis, four genes encode PDC, and the overexpression of *PDC1* and *PDC2* has been shown to improve survival under low‐oxygen conditions^8^, while the *pdc1* mutant is highly susceptible to anoxia^19^. However, not only *PDC1* but also *PDC2* is up-regulated when Arabidopsis plants are exposed to hypoxia^20^. *PDC1* and *PDC2* are both, independently, required for survival under low‐oxygen conditions, as they do not appear to be functionally redundant, since both the individual *pdc1* and *pdc2* mutants are more susceptible to hypoxia than the wild-type^20^. Interestingly, a double *pdc1pdc2* mutant appears to be considerably more susceptible to anoxia than the *adh1* mutant, which is in disagreement with the predominant role of ADH as a mechanism to regenerate NAD + from NADH generated along the glycolytic pathway. Higher susceptibility of the *adh1* mutant would be expected if acetaldehyde, which is toxic to plant cells^2^, accumulates in the *adh1* mutant, and without ADH activity NAD^+^ can only be regenerated by lactate dehydrogenase (LDH), which likely leads to cytoplasm acidosis^2^.

Although both *PDC1* and *PDC2* are strongly upregulated under hypoxia, their expression is already high under aerobic conditions^20^. Both PDC and ADH protein levels are also quite high in roots, even under aerobic conditions, while only PDC is clearly detectable in aerobic leaves^20^. The high level of PDC protein observed by immunoblotting in aerobic tissues raises the question of its metabolic role. In roots, the occurrence of hypoxic niches as a consequence of soil compactness may allow the expression of both ADH and PDC. On the other hand, in the aerobic leaves, where only PDC is expressed, PDC could act as a by‐pass for the activity of pyruvate dehydrogenase (PDH) as proposed by Tadege et al.^21^. PDC activity, coupled with aldehyde dehydrogenase (ALDH) activity, which is also constitutively present in aerobic leaves^19^, may prevent cellular build‐up of excess pyruvate, which might negatively affect respiration^22^.

The existence of hypoxic niches in otherwise fully aerobic tissues was recently described^23^. When plant shoot meristems develop, they become embedded in a low-oxygen niche, and hypoxia is required to regulate the production of new leaves^24^. Furthermore, hypoxic niches are established during lateral root primordia development in *Arabidopsis thaliana* grown under aerobic conditions, contributing to the downregulation of key auxin-induced genes and thus promoting the production of lateral roots^25^.

Hypoxic niches can also originate from plant–microbe interactions. Hypoxia-responsive genes are up-regulated in roots infected by *Plasmodiophora brassicae*^26^ or upon *Agrobacterium tumefaciens* infection of stems^27^, possibly resulting from a slower oxygen diffusion rate in tumorigenic tissues^28^ or from competition for oxygen between the pathogen and the host^26^. Infection of aerobic leaves of Arabidopsis by *Botrytis cinerea* and *Alternaira brassicicola* results in local hypoxia at the site of infection, probably because of increased respiration in the infected tissue^29^.

The importance of genes induced by hypoxia may thus go well beyond their classical role during environmental hypoxia due to waterlogging or flooding. Local oxygen availability in meristems likely limits respiration, and ATP production is likely limited to glycolysis coupled with fermentation^23^. *ADH1* and *PDC1* are expressed in the shoot apical meristem and in lateral root primordia (LRP)^24,25^, suggesting that fermentation may occur in plants growing in a normal aerobic environment. This would seem to indicate that deficiency in either ADH or PDC may affect the aerobic growth of plants.

Such evidence would help in answering the unsolved issue of metabolism in the hypoxic niches of aerobic plants^23^. However, to our knowledge there are no reports of reduced aerobic growth of defective mutants in either *ADH* or *PDC*. The only exception would seem to be a study by Minervini et al.^30^ performed with image-analysis assisted phenotyping, in which aerobically grown *adh1* Arabidopsis plants were found to be smaller than the wild-type^30^. However, their aim was to validate a new phenotyping platform rather than to explore the possible impact of defective fermentative metabolism^30^.

We therefore decided to perform a detailed analysis of the importance of ADH and PDC in Arabidopsis plants grown under three different conditions, namely aerobic conditions, waterlogging for a long time (19 days) and submergence for a relatively short period (35 h). The results, surprisingly, showed that the absence of functional ADH or PDC has a stronger impact in aerobic plants rather than on tolerance to environmental hypoxia.

## Results

### Development and optimization of image analysis procedures for phenotyping plants under hypoxia

Plant growth of wild-type (Col-0), *adh1* and *pdc1pdc2* plants did not reveal any obvious phenotype upon visual inspection. We therefore adopted a digital phenotypic approach using a LabScanalyzer (Lemnatec GmbH, Aachen, Germany), which consists of a cabinet containing a top-view high-resolution RGB camera and LED illumination. The software enables custom-made workflows to be run for growth analysis. A workflow consists of three main elements: i) image acquisition, ii) image processing, and iii) data aggregation (see Fig. S1).

**Figure 1:**
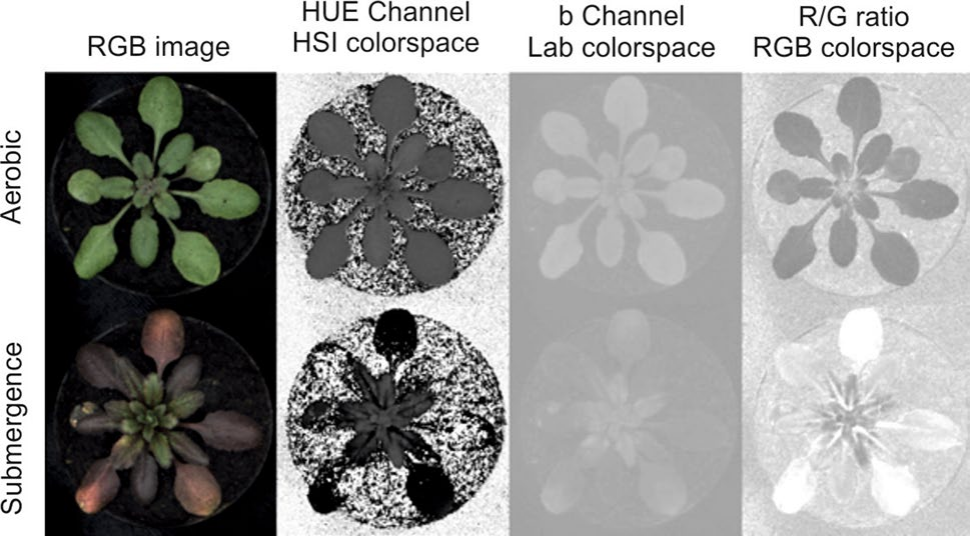
Arabidopsis grown under aerobic and submerged conditions to illustrate the reduced color contrast arising from the stress treatment in the HUE channel (HSI colorspace) b channel (Lab colorspace) and the Red/Green ratio (RGB colorspace). While the aerobic plant shows color contrast to the background, this contrast is greatly reduced for the submerged plant.

**Figure 2:**
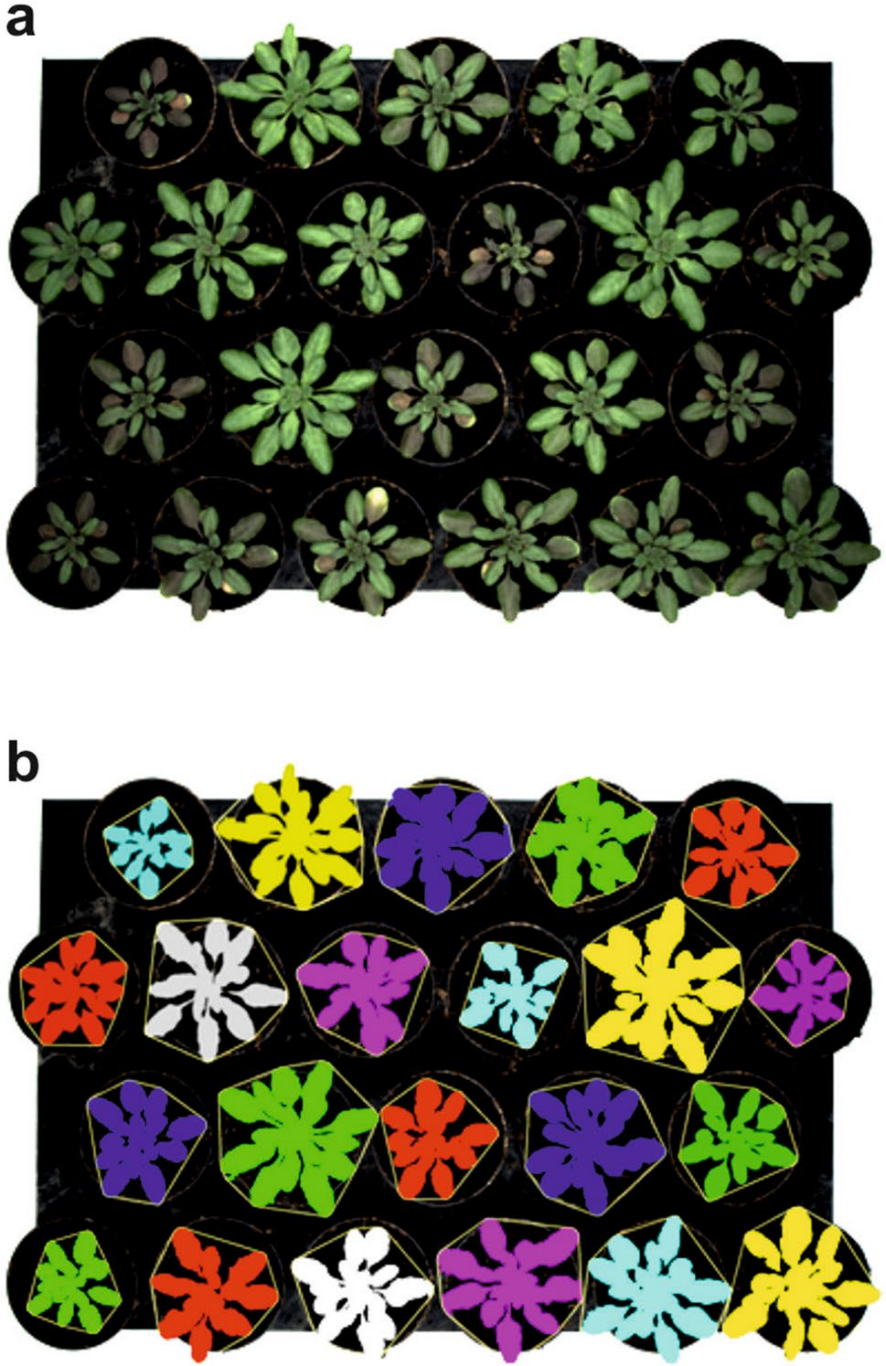
Image segmentation separating plants with green or reddish leaves from brownish background. Plants with reddish leaves are difficult to identify correctly using a phenotyping instrument based on RGB imaging unless training is performed and specific machine-learning algorithms are used. (**a**) Tray with Arabidopsis plants with different leaf colors. (**b**) Segmented image obtained with the software developed here, showing that almost all pixels are correctly associated with plants, regardless of their leaf color. Pixels related to each plant are colored as overlay on the original image. The convex hull area of the plants is also shown as a thin yellow line around the plant shape.

The leaves of Arabidopsis plants stressed by submergence or waterlogging changed to red and brown (Fig. 1). In this case, image segmentation (separation of plant and background pixels) based on colour features did not yield satisfactory results, because of the low colour contrast of leaves with the brownish soil (Fig. 1). We therefore followed a supervised pixel-based machine-learning segmentation approach using the Boosted Trees method. Six representative images of Arabidopsis trays were labelled using the freeware software GIMP (https://www.gimp.org/) resulting in a training data set of 292,769 background and 166,251 plant pixels. To train the algorithm, we used two colours (Red and Green channels), haar-like features (Table S1) and texture features (Table S2) from the OpenCV library. Eighty percent of the labeled data was used for training and 20% for evaluation. Background to plant pixel class balancing was applied. The evaluated prediction accuracy of the algorithm was 97.8%. The parameters of the Boosted Trees are given as a Supplemental file (Table S1, S2, as direct export of the OpenCV library). Further post-processing steps were followed, as illustrated in Fig. S1. Figure 2 shows one original and classified image after the use of the optimized Boosted Trees algorithm, which accurately identifies the size and shape of a plant even when the plant’s colour is very similar to the background.

### High-resolution RGB camera-assisted analysis reveals new phenotypic traits of adh1 and pdc1pdc2 mutants under aerobic and waterlogging conditions

**Figure 3:**
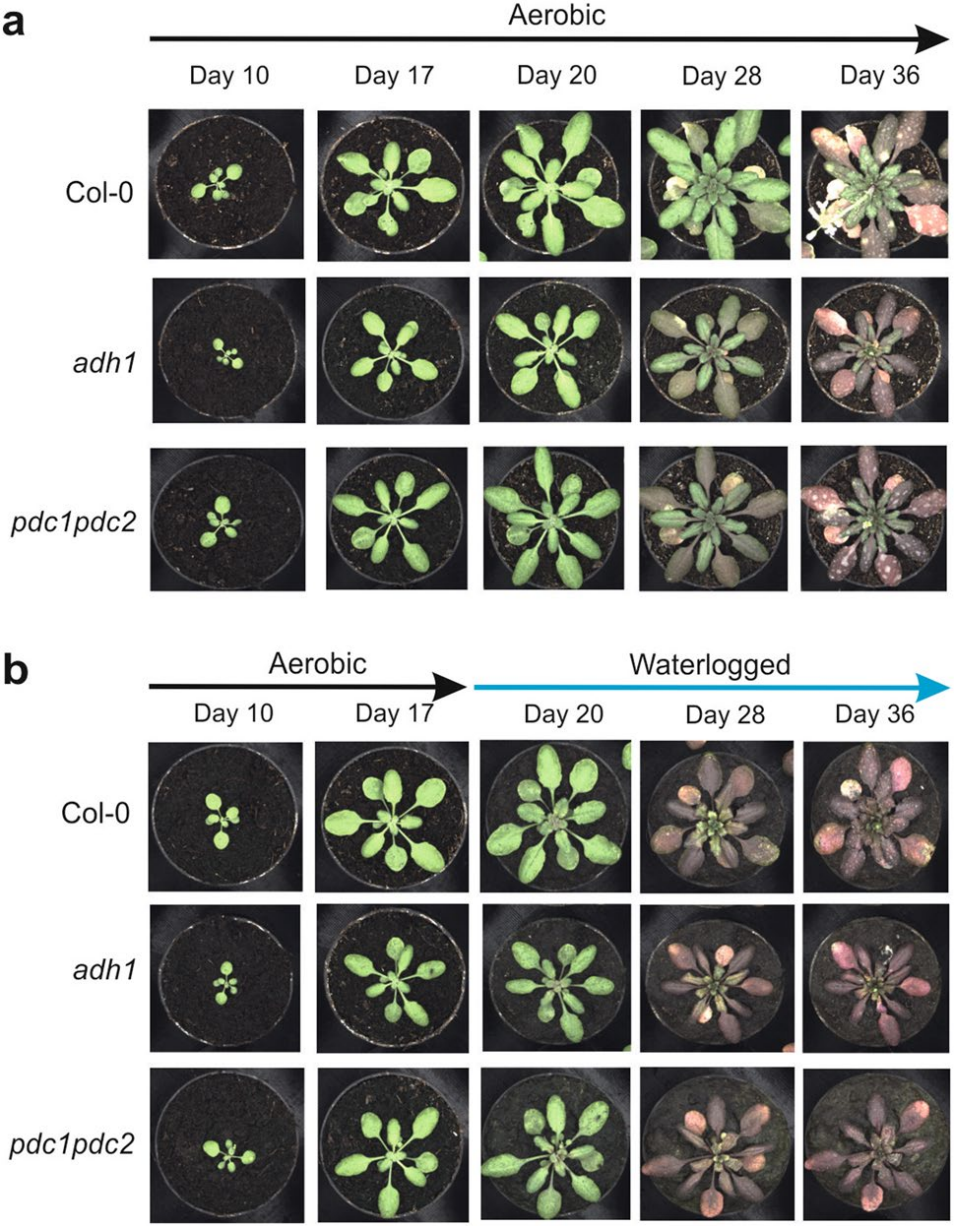
Effect of waterlogging on the phenotype of Arabidopsis plants. Plants were grown under aerobic conditions for 17 days and then transferred to waterlogging conditions. Waterlogging treatments were performed with plants having all the root system under water, but with the petioles and leaves above the water level. (**a**) Plants grown under aerobic conditions for 36 days. (**b**) Plants grown under aerobic conditions for 17 days and transferred to waterlogging conditions for up to 36 days.

**Figure 4:**
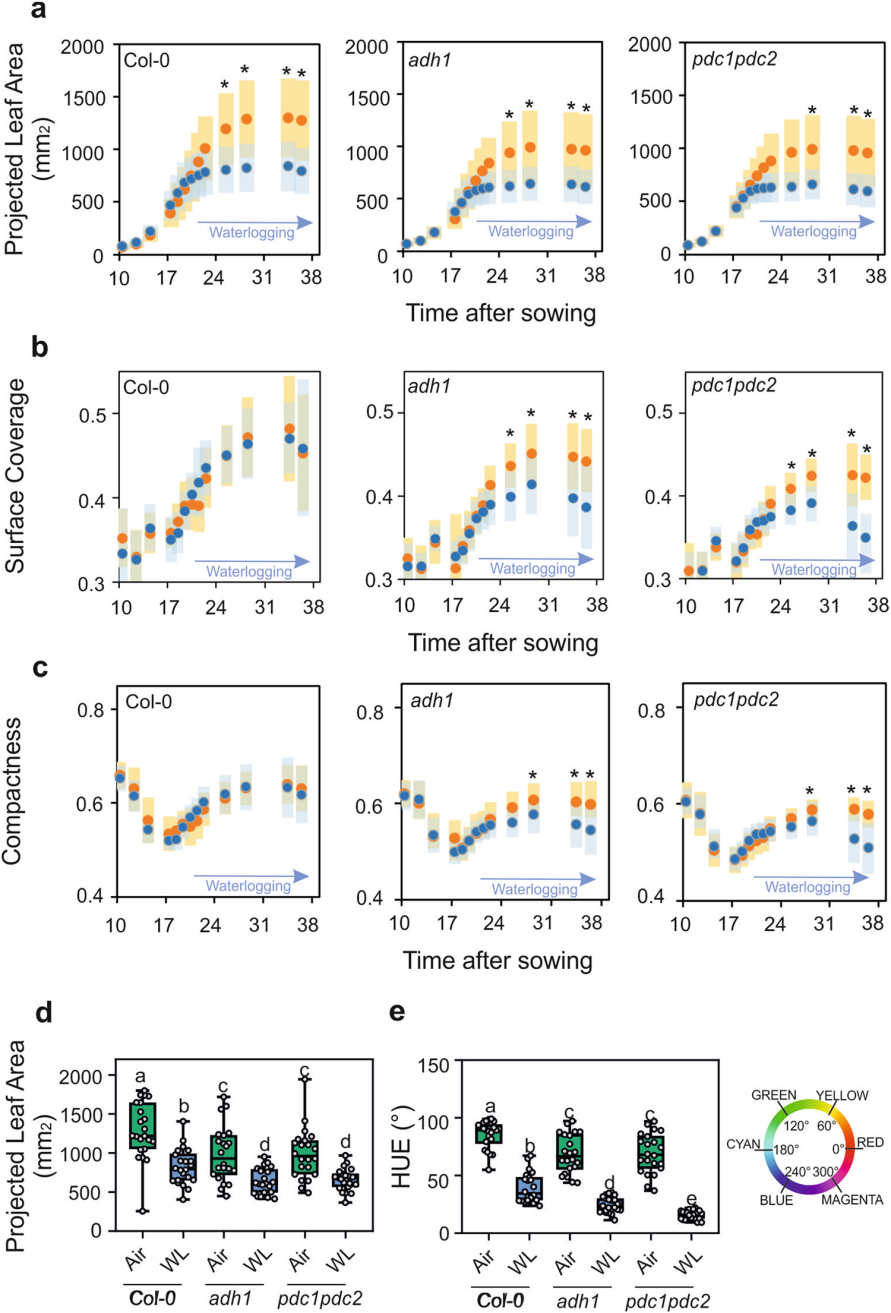
Plant size (**a**) and morphology traits (**b, c**) plotted against time in aerobic plants (orange symbols) compared to plants that were waterlogged (blue symbols). Variance (± SD, n = 22) is shown as color-shaded areas (yellow: aerobic; light blue: waterlogging). Statistically significant differences are indicated by an asterisk (*T* test, pairwise comparison at each time point, * = *p* < 0.01). (**d**) Plant size (projected leaf area) in aerobic plants (green box) compared to plants that were waterlogged (blue box). Data were taken at day 28. (**e**) Color (HUE-value from HSI color space) in aerobic plants (green box) compared to plants that were waterlogged (blue box). The color wheel gives values for HUE ranging from 0 to 360°. Data were taken at day 28. Lines in the boxes indicate the median. The bottom and top of each box denote the first and third quartile, respectively. The dots represent the single data points and whiskers denote the min/max values. Different letters indicate statistically significant differences (ANOVA).

Wild-type plants were subjected to a long period of waterlogging during which the root system was constantly submerged, but the leaves and petioles were above the water level (Fig. 3). The wild-type waterlogged plants displayed a strong reduction in growth as assessed by the projected leaf area parameter (PLA), representing a good index of plant size (Fig. 4a). A similar reduction in size was observed in *adh1* and *pdc1pdc2* plants (Fig. 4a). Analysis of surface coverage (the ratio of leaf area to the area of the minimum enclosing circle calculated from the top view image) showed that this morphological parameter does not change in the wild-type as a consequence of waterlogging (Fig. 4b), but it was severely affected in both *adh1* and *pdc1pdc2* plants. Likewise, compactness, which measures the degree of leaf area coverage (rosette area/conhull area), as with the agronomic measure of Leaf Area Index (LAI) was significantly affected by waterlogging in both *adh1* and *pdc1pdc2* but not in the wild-type plants (Fig. 4c).

Statistical analysis of the PLA on 28 day-old plants revealed that not only did the waterlogging significantly affect the plant size, but it also highlighted the different sizes of the three genotypes, even under aerobic conditions, with both *adh1* and *pdc1pdc2* plants being significantly smaller (Fig. 4d). An independent experiment confirmed these findings (Fig. S2a). The colour of Arabidopsis plants subjected to waterlogging changed quite significantly, turning from green to red shades (Fig. 3). The change in the hue was measured and the data showed that the *adh1* and *pdc1pdc2* plants already display a slight change in intensity when aerobic, but the shift towards red was evident in waterlogged plants which became more red, though with differences among genotypes, with the fermentation-defective mutants displaying a stronger reduction in the hue value (Fig. 4e).

### Short-term submergence is more detrimental than long-term waterlogging

**Figure 5:**
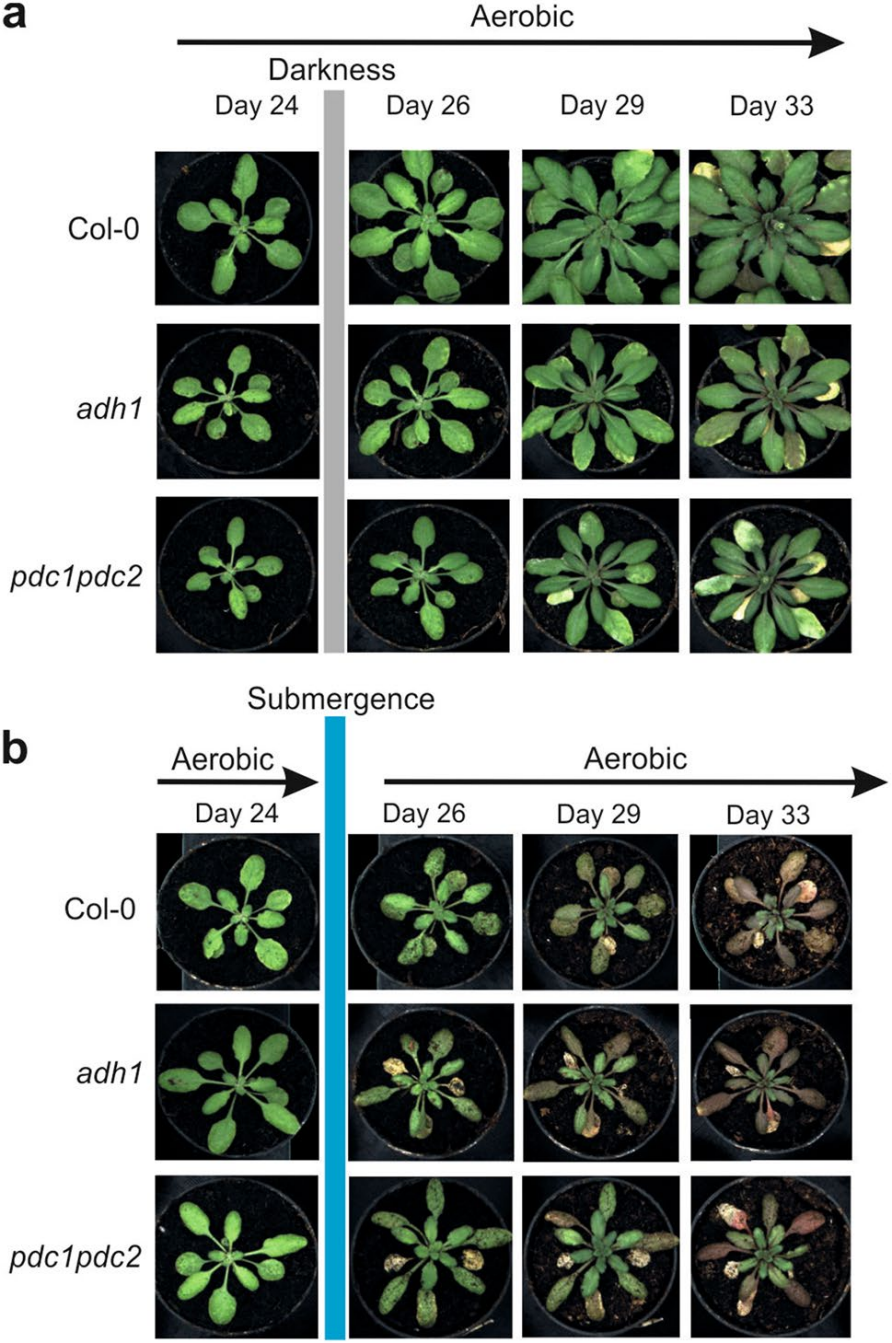
Effect of submergence on the phenotype of Arabidopsis plants. Plants were grown under aerobic conditions for 24 days and then transferred to submergence for 35 h. Plants were then transferred back to aerobic conditions. Tanks with a water level of 10 cm above leaf level were used to submerge the plants. (**a**) Plants grown under aerobic conditions for 33 days. (**b**) Plants grown under aerobic conditions for 24 days, and transferred to submergence for 35 h, and back to aerobic conditions until day 33.

**Figure 6:**
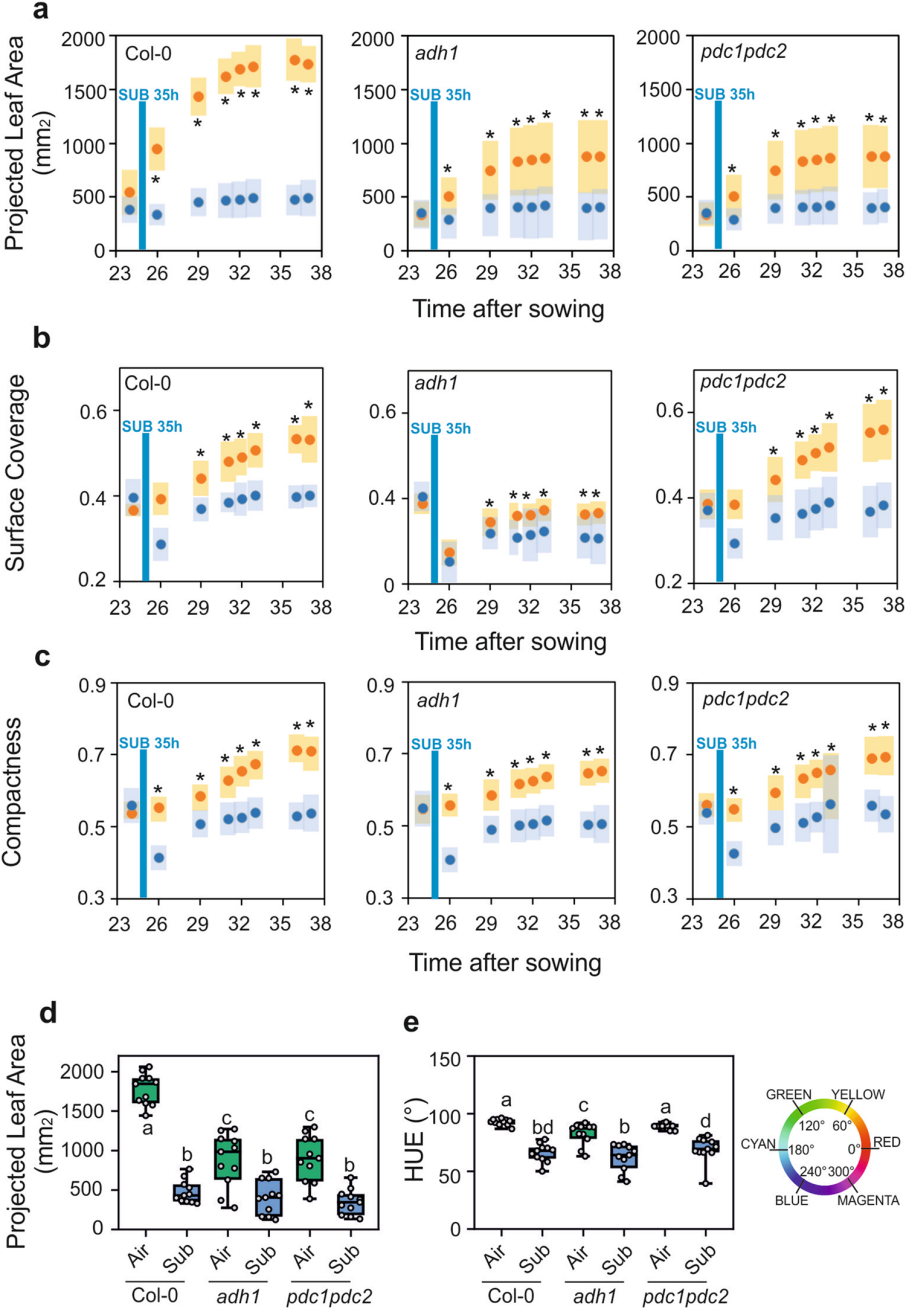
Plant size (**a**) and morphology traits (**b**, **c**) plotted against time in aerobic plants (orange symbols) compared to plants that were submerged for 35 h (blue symbols). Variance (± SD, n = 22) is shown as color-shaded areas (yellow: aerobic; light blue: submergence). Statistically significant differences are indicated by an asterisk (T-test, pairwise comparison at each time point, *p* < 0.01). (**d**) Plant size (projected leaf area) in aerobic plants (green box) compared to plants that were submerged (blue box). Data were taken at day 33. (**e**) Color (HUE value; HSI color space) in aerobic plants (green box) compared to plants that were submerged (blue box). The color wheel shows values for HUE ranging from 0 to 360°. Data were taken at day 33. Lines in the boxes indicate the median. The bottom and top of each box denote the first and third quartile, respectively. The dots represent the single data points and whiskers denote the min/max values. Different letters indicate statistically significant differences (ANOVA).

The impact of 35 h of submergence on Arabidopsis plant growth was quite strong, with plants of the three genotypes responding by almost stopping growth (Fig. 5). Both *adh1* and *pdc1pdc2* plants grew slower than the wild-type under aerobic conditions, confirming the dataset collected with aerobic plants in the waterlogging experiment (Fig. 6a). Surface coverage and compactness were affected by submergence in *adh1* and *pdc1pdc2* plants, indicating that these two morphological parameters are associated with the absence of ADH and PDC in both waterlogged and submerged plants (Fig. 6b,c).

Statistical analysis of 33 day-old plants showed a clear difference in the size (PLA) of the two mutants compared to the wild-type in aerobic conditions (Fig. 6d). An independent experiment confirmed these findings (Fig. S2b). These results highlighted the importance of ADH and PDC in aerobic plant growth. Moreover, the growth of all the genotypes was severely reduced as a consequence of submergence, although the percentage reduction was much higher in the wild-type compared to both *adh1* and *pdc1pdc2* plants (Fig. 6d). Submergence led to a change in hue (Fig. 6e), which when measured revealed that all the genotypes responded similarly.

### ADH1 and PDC1 are expressed in aerobic roots and strongly upregulated by waterlogging and submergence

**Figure 7:**
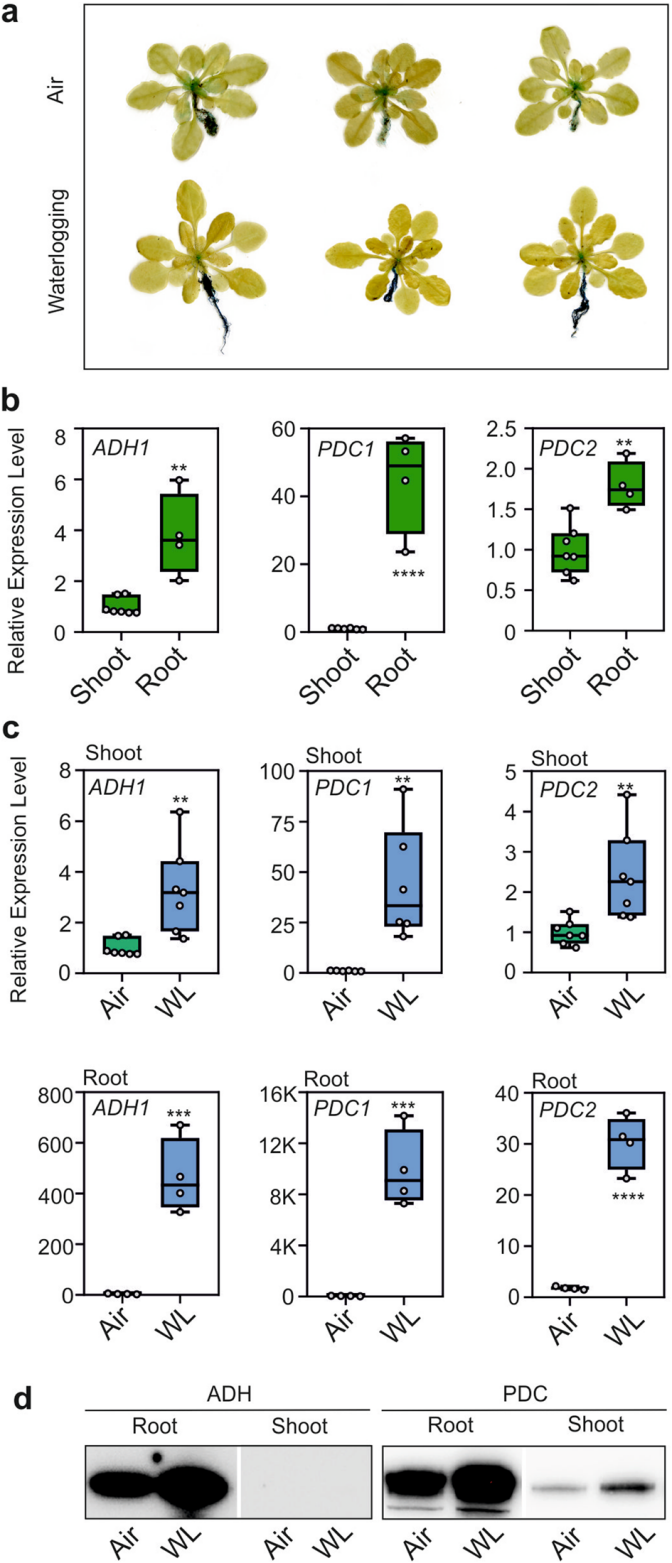
Expression of *ADH1* and *PDC1* in aerobic and waterlogged Arabidopsis plants. (**a**) GUS staining of *pADH1:GUS* plants in air or 5-day waterlogging. (**b**) Comparison of the expression (RT-qPCR) of *ADH1*, *PDC1*, and *PDC2* in roots and shoots of aerobic plants. (**c**) Comparison of the expression (RT-qPCR) of *ADH1*, *PDC1*, and *PDC2* in air (green box) vs. 5-day waterlogging (WL, blue box). Lines in the boxes indicate the median. The bottom and top of each box denote the first and third quartile, respectively. The dots represent the single data points and whiskers denote the min/max values. Statistically significant differences are indicated by an asterisk (*T* test, pairwise comparison at each time point, * = *p* < 0.05; ** = *p* < 0.01; *** = *p* < 0.001; **** = *p* < 0.0001). (**d**) Immunoblot analysis of proteins in aerobic and waterlogged plants. Equal loading was verified by protein staining (Figure S4).

Given the reduction in growth observed in *adh1* and *pdc1pdc2* plants, even when kept under aerobic conditions, we verified the expression of *ADH1* and *PDC1* by reporter gene expression (*pADH1:GUS*) and qPCR. Expression of *ADH1* in the aerobic plants was evident in the root system and in the younger leaf tissues (Fig. 7a). Waterlogging moderately increased the GUS staining in the root system (Fig. 7a). These results were confirmed using two independent *pADH1:GUS* lines (Fig. S3). qPCR analysis of mRNA level revealed a higher level of expression of *ADH1*, *PDC1* and *PDC2* in the roots compared to the shoots (rosettes) in aerobic conditions (Fig. 7b). Waterlogging resulted in a dramatic increase in the expression of *ADH1*, *PDC1* and *PDC2* and, interestingly, also in the shoot, which is itself aerobic, given that only the root system is under water (Fig. 7c).

To investigate whether ADH and PDC proteins were actually synthesized during waterlogging, we performed a western-blot analysis (Fig. 7d). In roots, both ADH and PDC proteins were already present in aerobic tissues and were induced further by waterlogging. In shoots, only PDC was detected under normoxia. It was further induced during the stress (Fig. 7d). We also assayed ADH activity and the results showed that in the roots, the protein level was already present under air conditions and was induced during waterlogging (Fig.S5), confirming the western-blot results.

Genes encoding for enzymes involved in ethylene synthesis were also upregulated, above all in the waterlogged roots, suggesting that ethylene or its precursor 1-aminocyclopropane-1-carboxylic acid (ACC) may represent root-to-shoot signaling molecules triggering the induction of *ADH1* and *PDC1* in the shoots of waterlogged plants (Fig. 7c). 1-aminocyclopropane-1-carboxylic acid synthase 2 (*ACS2*) and 1-Aminocyclopropane-1-carboxylic acid oxidase 1 (*ACO1*) are hypoxia-reponsive^31^ and were strongly induced in the roots of waterlogged plants (Fig. S6a). This hypothesis, however, was dismissed by the fact that the induction of *ADH1* and *PDC1* in the shoots of waterlogged plants was retained in two ethylene insensitive mutants (*ein2-1* and *etr1-3*) (Fig. S6b,c).

**Figure 8:**
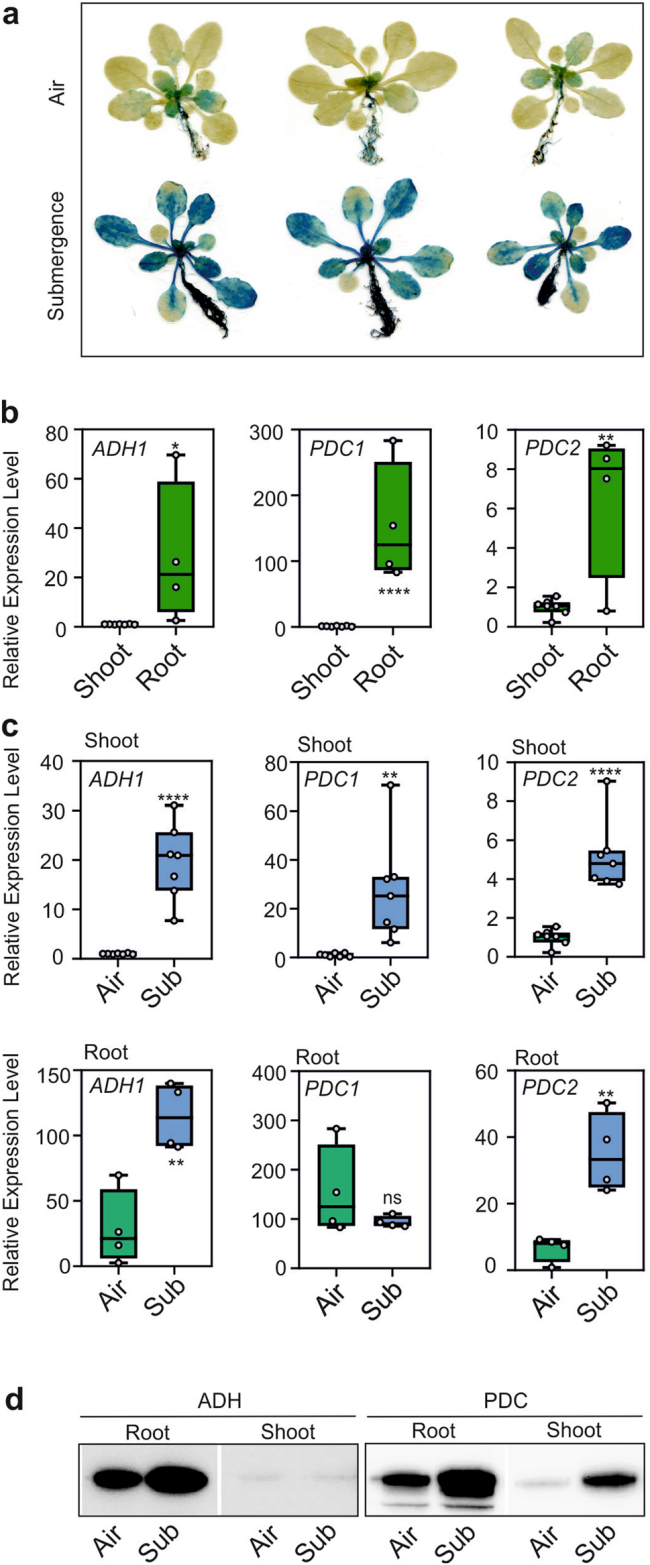
Expression of *ADH1*, *PDC1*, and *PDC2* in aerobic and submerged Arabidopsis plants. (**a**) GUS staining of pADH1:GUS plants in air or 35 h submergence. (**b**) Comparison of the expression (RT-qPCR) of *ADH1* and *PDC1* in roots and shoots of aerobic plants. (**c**) Comparison of the expression (RT-qPCR) of *ADH1*, *PDC1* and *PDC2* in air (green box) vs. 35 h submergence (Sub, blue box). Lines in the boxes indicate the median. The bottom and top of each box denote the first and third quartile, respectively. The dots represent the single data points and whiskers denote the min/max values. Statistically significant differences are indicated by an asterisk (T-test, pairwise comparison at each time point, * = *p* < 0.05; ** = *p* < 0.01; *** = *p* < 0.001; **** = *p* < 0.0001). (**d**) Immunoblot analysis of proteins in aerobic and submerged plants. Equal loading was verified by protein staining (Figure S4).

The impact of submergence of the whole plant on the expression of *pADH1:GUS* was strong, with almost all plant organs displaying expression of the GUS reporter (Fig. 8a). Aerobic plants displayed GUS activity in the root system and younger leaves (Fig. 8a). *ADH1*, *PDC1* and *PDC2* were already expressed in aerobic roots compared to shoots (Fig. 8b). These results were confirmed using two independent *pADH1:GUS* lines (Fig. S7). *ADH1* was upregulated in both shoots and roots by submergence, while the expression of *PDC1* and *PDC2* increased after submergence in shoots (Fig. 8c). In roots only *PDC2* expression increased after submersion while *PDC1* is already high in air (Fig. 8b,c). As described above for the waterlogging experiment, the western-blot showed that in both tissues, ADH and PDC were already present in aerobic conditions but were induced under submersion, with the exception of ADH in shoots (Fig. 8d). ADH activity data showed that its activity was induced both in roots and shoots (Fig S5).

Submergence exerted a modest effect on ethylene-biosynthesis genes, with only *ACS2* showing induction after shoot submergence (Fig. S8a). As previously shown in the waterlogging experiment, ethylene-signaling mutants showed the same level of induction of *PDC1* and *ADH1* (Fig. S8b,c), thus ruling out a role for ethylene under these experimental conditions.

## Discussion

The existence of hypoxic niches in otherwise fully aerobic tissues suggests a possible role for the fermentative metabolism in plants grown under what appear to be aerobic conditions. Hypoxia is detected in bulky organs such as tubers and fruits^32^, and in the phloem^33^ with hypoxia occurring as a constitutive condition of specific tissues in which oxygen gradients act as a regulatory cue^24,25^. It was demonstrated that a protein that is required for setting the leaf production rate, namely LITTLE ZIPPER 2 (ZPR2), is highly unstable in aerobic tissues, but stabilizes if the tissue is hypoxic^24^. This is because ZPR2 possesses an oxygen-sensitive penultimate N-terminal Cys residue (Cys2), which can be exposed at the N terminus by removing the initial methionine by a Met aminopeptidase^34^. Under aerobic conditions, Cys can be oxidized by plant cysteine oxidases^35^. Oxidation of Cys channels the protein to ubiquitination and subsequent proteasomal degradation, thus following the N-degron pathway^36^. Although ZPR2 does not actually play a role in the response of plants to low oxygen, its stability in the SAM highlights that the meristem is hypoxic, and the energy demand in hypoxic niches is thus likely met via ATP production by glycolysis coupled with fermentation^37^.

*ADH* and *PDC* are highly expressed in the SAM and LRP^24,25^. Although glycolysis coupled with fermentation produces fewer ATP molecules per unit of glucose compared to oxidative phosphorylation^2^, glycolysis produces ATP at a faster rate when reserves are adequate^23^. Given the crucial role of both PDC and ADH in facilitating the activity of glycolysis in the absence of the NADH re-oxidizing activity of the electron transport chain in the mitochondria, the ability of the plant to grow is likely affected if the anaerobic metabolism in the SAM, LRP and other anaerobic tissues in otherwise aerobic plants is compromised.

The availability of mutants that are blocked in their ability to carry out fermentation enabled us to test this hypothesis. The two mutants, namely the *adh1* mutant and the double *pdc1pdc2* mutant, are both unable to carry out ethanol production. Although ADH is the crucial enzyme for NADH oxidation, mutations in ADH can also lead to the accumulation of acetaldehyde, which is much more toxic than ethanol^38^. The *pdc1pdc2* is instead blocked at the level of pyruvate, and therefore, while the two mutants are both equally impaired in their ability to produce ethanol^39^, they differ in terms of the metabolic step blocked and the possible detrimental effects of acetaldehyde accumulating in the *adh1* mutant could be revealed by differences in the phenotypes of the two mutants.

The growth comparison of the wild-type and mutants entailed the semi-automatic phenotyping of Arabidopsis plants in order to reveal the specific phenotype of plant exposed to environmental hypoxia. We compared the three genotypes under three environmental conditions, two of which restricted oxygen availability due to either long-term waterlogging or short-term submergence. Under these conditions the colour of the plant rapidly changed to shades of red, making it very difficult for computer-assisted image analysis to distinguish the plant from the brownish soil in the background (Fig. 1). We therefore used a supervised pixel-based machine-learning approach that automatically identified the plant shape (Fig. 2), and in just a few minutes we collected a vast number of morphological parameters from a relatively large number of replicates. Machine-learning based segmentation approaches can be easily applied to different cases by training them with new labelled images. Nonetheless the light and camera settings in an imaging cabinet should be reproducible in order to reduce the size of the training data set required.

RGB camera-assisted phenotyping revealed the differences between the three genotypes, in terms of growth parameters, under aerobic and low oxygen conditions. The differences in plant size observed when comparing aerobic plants were larger than those in oxygen deprived plants (Figs. 4 and 6). Although differences between the wild-type and the mutants were observed in terms of plant size (PLA) in waterlogged plants, there was no PLA difference when comparing the three genotypes that had undergone short-term submergence.

These results indicated that the growth penalty associated with the lack of fermentative metabolism is much greater in plants grown under aerobic conditions than in oxygen-deprived environments. The differences in PLA and the morphology of mutants affected in fermentative metabolism when compared to the wild-type suggest that this pathway is active and important for cell functionality in hypoxic niches in plants. On the other hand, the small difference observed in oxygen deprived environments suggests that, besides fermentation, other metabolic pathways or responses might be more relevant for tolerance to environmental hypoxia. The induction of a wide number of mRNAs in plants subjected to hypoxia suggests that the adaptive response to the lack of oxygen goes well beyond the adaptation of energy metabolism through glycolysis coupled with fermentation. In fact, the hypoxia-induced mRNAs include conserved responses associated with glycolysis and fermentation, but also with alternative respiration, metabolite transport, reactive oxygen species amelioration, chaperone activity, and ribosome biogenesis^40^.

Soil compactness may generate hypoxic conditions in the root system, even in the absence of excess water (waterlogging). Our results showed that the root system is hypoxic, even under lab conditions using a very well aerated potting medium. It would therefore be expected that under natural conditions, hypoxic conditions may be very easily encountered by roots. This may influence the response in the plant’s aerial tissues, since waterlogging led to the induction of *ADH1* and *PDC1* in the aerobic shoot as well (Fig. 7C).

Ethylene production is observed in plants that are waterlogged^41^ and ethylene or its precursor ACC has been proposed as a root-to-shoot signaling molecule^42^^.^ Furthermore, ethylene can enhance ERFVII protein stability prior to hypoxia by increasing the NO-scavenger PHYTOGLOBIN1, thus pre-adapting plants to survive subsequent hypoxia (ethylene priming)^31,43^. However, we found no link between ethylene and the induction of *ADH1* and *PDC1* in the aerial organs of waterlogged plants. In any case, waterlogging represents a long-lasting physiological constraint to plants, and we did not expect ethylene priming to exert its effects under these conditions. The nature of the signaling that triggers the induction of *ADH1* and *PDC1* in the shoots of waterlogged plants therefore remains to be identified. The induction of *ADH1* and *PDC1* in aerobic tissues such as the shoots of waterlogged plants reinforces the importance of fermentation being active in otherwise aerobic tissues, thus highlighting that these enzymes are involved in more than just abiotic stress conditions.

Computer-assisted phenotyping can reveal morphological parameters that are otherwise hardly detectable by the human eye. Here, we demonstrated that this technology, when properly fine-tuned to fit the particular color characteristics of Arabidopsis plants exposed to low oxygen conditions, can detect several traits that have never been reported before for mutants affected in the fermentative pathway. Besides plant size (PLA), differences in surface coverage and compactness were observed.

Our results highlight the previously underestimated role of fermentation under aerobic conditions and go some way to explaining why plants developed a very sophisticated mechanism for oxygen sensing upstream of the induction of anaerobic genes such as *ADH1* and *PDC1*. Environmental hypoxia is an occasional stress for most plants, while hypoxic niches are likely present in every higher plant species^24^, imposing quite a strong evolutionary pressure for the development of a complex and efficient mechanism to sense and adapt to hypoxia^32^.

We thus hypothesize that constitutive hypoxic niches in aerobic multicellular plants, rather than occasional waterlogging events, drove the evolution of their highly sophisticated oxygen sensing machinery.

## Methods

### Plant material and growth conditions

Genotypes of Arabidopsis (*Arabidopsis thaliana*) used included the Col-0 ecotype, the *adh1* (N552699) mutant and the double mutant *pdc1pdc2* (N660027crossed with N862662) mutant^39^. The N552699 line (*adh1*) was compared with an independent *adh1* mutant (R002), revealing that they have a large number of physiological parameters in common^22^. N552699 was also recently verified^39^. The *pdc1* and *pdc2* mutant were previously described^20^ and the cross *pdc1pdc2* was described and verified^39^. The *etr1–3* (N66985), and *ein2–1* (N65994) seeds were obtained from the Nottingham Arabidopsis Stock Center. Plants were grown in pots (70% peat-moss based professional potting medium with 30% perlite) for 3–4 weeks at 23 °C with a 12/12-h photoperiod at 120 μmol photons m^−2^ s^−1^ before being used in our experiments. Extreme care was taken to prevent the over-watering of plants. Waterlogging treatments were performed on 3-week old plants, during which all the root system was immersed in water, but the leaves and petioles were above the water level. For the submergence experiments, the plants were submerged in tanks with a water level of 10 cm above leaf level. Oxygen concentration was measured in the tanks used for submerging the plants with a FireStingO2 high precision, personal computer–controlled fiber-optic oxygen meter (Pyro Science). The oxygen probe used was OXROB10. Water temperature was measured using the Dipping-Probe Temperature Sensor TDIP15. The oxygen levels measured were as previously reported^44^. The submergence treatment was carried out in the dark (also the controls in air were in the dark), while waterlogged plants were kept under the normal day/night cycle. Plant trays were placed in a LabScanalyzer (LemnaTec, GmbH, Aachen, Germany) for the acquisition of images.

### Image acquisition

Plant trays were placed in a LabScanalyzer (LemnaTec, GmbH, Aachen, Germany) imaging box equipped with a Manta G-1236 camera (Allied Vision Technologies GmbH, Stadtroda, Germany) and a Kowa LM12XC lens (Kowa Optimed Deutschland GmbH, Düsseldorf, Germany). Trays were illuminated by two cool white LED panels (polyscale GmbH & Co. KG, Aachen, Germany) mounted beside the camera at an angle of 30° to prevent direct reflection from the imaging area. The raw images acquired were demosaiced using the AHD (Adaptive Homogeneity-Directed Demosaicing) algorithm from the OpenCV library and stored as 8 bit PNG images. Images were analysed with LemnaGrid software (LemnaTec, GmbH, Aachen) resulting in one text file (csv), which was stored using the same filename as the image (see Figure S1 showing the data flow scheme). See Figure S9 for a description of the phenotypic parameters measured.

### Generation of transgenic lines

The *ADH* promoter was amplified with Phusion High Fidelity DNA-polymerase (Thermo-Fischer Scientific) using pADH_Fw (ggggagctcttcacacaacacactgaag) and pADH_Rv (tatcaacagtgaagaacttgcactagtccc). The DNA fragment obtained was cloned into the pH7WG2 plasmid^45^ using SacI and SpeI restriction sites. The generated pH7WGpADH plasmid was recombined using a GatewayTM LR ClonaseTM II Enzyme mix (Thermo-Fisher Scientific) with the β-Glucuronidase (GUS) entry vector (Thermo-Fischer Scientific). Transgenic Arabidopsis plants were obtained by Agrobacterium-mediated transformation, applying the floral-dip method^46^. Seeds were sterilized with 70% ethanol and 10% commercial bleach solution, and then rinsed five times with sterile distilled water. Seeds were selected on solid MS1/2 media supplemented with Hygromycin B (Duchefa) 50 µg/ml. Positive seedlings were moved to soil and transgene presence was assessed by PCR and GUS staining.

### RNA Extraction and qPCR analysis

Total RNA was extracted as previously described^29,47^ with a minor modification (omission of aurintricarboxylic acid) to make the protocol compatible with the subsequent PCR procedures. Electrophoresis using a 1% agarose gel was performed for all RNA samples to check for RNA integrity, followed by spectrophotometric quantification.

cDNA was synthesized as previously described^29^. Briefly, 1 mg of total RNA was retrotranscribed using the Maxima Reverse Transcriptase kit (Life Technologies). qPCR amplification^29^ was performed on 30 ng of cDNA with a ABI Prism 7300 sequence detection system (Applied Biosystems), using the PowerUp SYBRGreen Master Mix (Applied Biosystems). Ubiquitin10 (At4g053290) was exploited as the housekeeping gene^29^. Relative expression levels were calculated using GeNorm (https://genorm.cmgg.be/). A full list of the primers used for qPCR is provided in Supplemental Table S3.

### GUS staining

Histochemical GUS staining was carried out according to Jefferson et al.^48^. Briefly, plant material was fixed immediately after sampling in ice-cold 90% acetone for 1 h, rinsed several times in 100 mM phosphate buffer (pH 7.2), and then stained in a freshly-prepared reaction solution (0.2% Triton X-100, 2 mM potassium ferrocyanide, 2 mM potassium ferricyanide, and 2 mM X-Gluc [5-bromo-4-chloro-3-indolyl β-d-glucuronide, sodium salt dissolved in DMSO] in 100 mM phosphate buffer, pH 7.2). Plants were stained overnight, and chlorophyll was eliminated from green tissues by washing them with 96% ethanol.

### Extraction of Proteins, SDS-PAGE, immunoblots

Plant material was extracted by grinding precooled samples in liquid nitrogen with a pestle to a fine powder^44^. The extraction buffer (50 mM Tris–HCl, pH 7, and 1% SDS with Sigma P9599 protease inhibitor cocktail) was added, vortexed vigorously, and then centrifuged for 30 min at 14,000 rpm to obtain a supernatant. Protein content in the supernatant was quantified with Bio-Rad DC reagent (Lowry method). Samples were dissolved in Laemmli buffer, treated at 95 °C for 10 min, and loaded (40 mg) onto Invitrogen NuPAGE gels (10%Bis–Tris Midi Gels). After electrophoresis, proteins were transferred to a PVDF membrane using the Bio-Rad Trans-Blot turbo transfer pack. An anti-ADH antibody (Agrisera AS10685, Agrisera, Vännäs, Sweden), an anti-PDC antibody (Agrisera AS10691; Agrisera, Vännäs, Sweden), and secondary goat anti-rabbit IgG HRP conjugated antibody (Agrisera AS09 602; Agrisera, Vännäs, Sweden) were used. Detection was performed using the LiteAblot TurboChemiluminescence substrate (Euroclone). Amido black staining was performed to check equal loading. The blot was stained for 10 min [0.1% amido black (Sigma Aldrich, Milan, Italy), 45% methanol, 10%acetic acid] and then washed in a destaining solution (90%methanol/2% acetic acid/8% water) for 2 min.

### Enzyme assays

Samples were rapidly frozen in liquid nitrogen, ground to a powder, and centrifuged. The supernatant was assayed for ADH activity in a 1 ml assay mixture (100 mM Tris–HCl pH 9, 5 mM DTT, 5 mM MgCl2, 0.5 mM NAD, 200 mM EtOH) monitoring the absorbance spectrophotometrically at 340 nm for 20 min. One unit is the amount of enzyme leading to a change in A_340_ of 0.1 in 1 min.

### Statistical analyses

Values that significantly differ from each other are indicated by different letters in figures (according to two-way ANOVA test, Benjamini and Hochberg False Discovery Rate post hoc test, *p* < 0.05) unless stated otherwise in the figures. Pairwise comparison was performed by a T-test.

## Supplementary information

Supplementary file1
