# Supplementary material for: Arabidopsis phenotyping reveals the importance of alcohol dehydrogenase and pyruvate decarboxylase for aerobic plant growth
Source: Sci Rep. 2020 Oct 7;10:16669. doi: 10.1038/s41598-020-73704-x (PMC7542448; doi:10.1038/s41598-020-73704-x)
Supplement: Supplementary file 1 — Supplementary file1 [file 41598_2020_73704_MOESM1_ESM.pdf]

Arabidopsis phenotyping reveals the importance of ALCOHOL DEHYDROGENASE and PYRUVATE DECARBOXYLASE for aerobic plant growth

Irene Ventura, Luca Brunello, Sergio Iacopino, Maria Cristina Valeri, Giacomo Novi, Tino Dornbusch, Pierdomenico Perata, Elena Loreti

Supplementary Information

Supplemental Table S1: List of haar-like features used for the machine-learning algorithm from the OpenCV library.

Color channel	Feature Name	Type	feature size	calculation
**Red**	haar_6	Block	8	Sum
**Red**	haar_8	Block	8	Sum
**Red**	haar_9	Block	8	Sum
**Red**	haar_10	Block	8	Sum
**Red**	haar_11	Block	8	Sum
**Red**	haar_12	Block	8	Sum
**Red**	haar_13	Block	8	Sum
**Red**	haar_17	Line	1x3	Sum
**Red**	haar_17	Line	4x12	Sum
**Green**	Haar_5	Block	15	Sum
**Green**	Haar_5	Block	30	Sum
**Green**	Haar_10	Block	15	Sum
**Green**	Haar_10	Block	30	Sum
**Green**	Haar_12	Block	15	Sum
**Green**	Haar_12	Block	30	Sum

1. **Supplemental Table S2:** list of texture features used following Laws 1980 (Laws, K. I. Textured image segmentation (No. USCIPI-940). University of Southern California Los Angeles Image Processing INST. (1980) taken from the OpenCV library.

Color channel	Feature name	Block size
**Red**	E5E5	15x15
**Red**	E5L5	15x15
**Red**	E5R5	15x15
**Red**	L5S5	15x15
**Red**	L5R5	15x15
**Red**	L5S5	15x15
**Red**	R5E5	15x15
**Red**	R5L5	15x15
**Red**	R5R5	15x15
**Red**	R5S5	15x15
**Red**	S5E5	15x15
**Red**	S5L5	15x15
**Red**	S5R5	15x15
**Red**	S5S5	15x15

Supplemental Table S3. List of primers.

Gene (AGI)	Forward primer	Reverse primer
At4g05320 (UBQ10)	GGCCTTGTATAATCCCTGATGAATAAG	AAAGAGATAACAGGAACGGAAACATAG T
At1g77120 (ADH)	CAATTGCTGGACTGCAAAGGTG	AGCAACTCTTTGCTCGTATCATCC
At4g33070 (PDC1)	CAATTGCTGGACTGCAAAGGTG	AGCAACTCTTTGCTCGTATCATCC
AT5G54960 (PDC2)	CCCCAAATCCGCAGTAGAGT	CCTCAAGGGGACACACATTT
AT1G01480 (ACS2)	ATGGGTCTTGCAGAGAATCAGC	ACCTTCAAGGGTGCAAATAGAAGC
AT2G19590 *(ACO1)*	ACCTCAGATGCAGATTGGGAAAGC	CCATCGTCTTGCTGAGTTCCTCTG

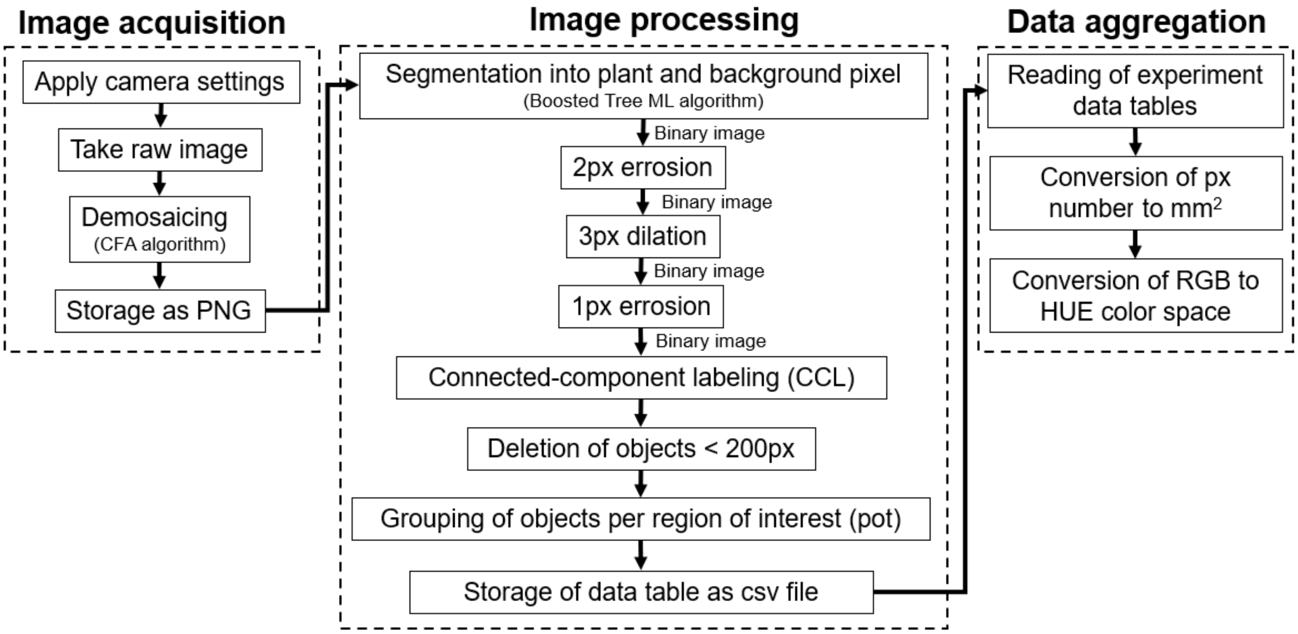

Figure S1. Scheme illustrating the main steps of a phenotyping workflow to generate standardised output results.

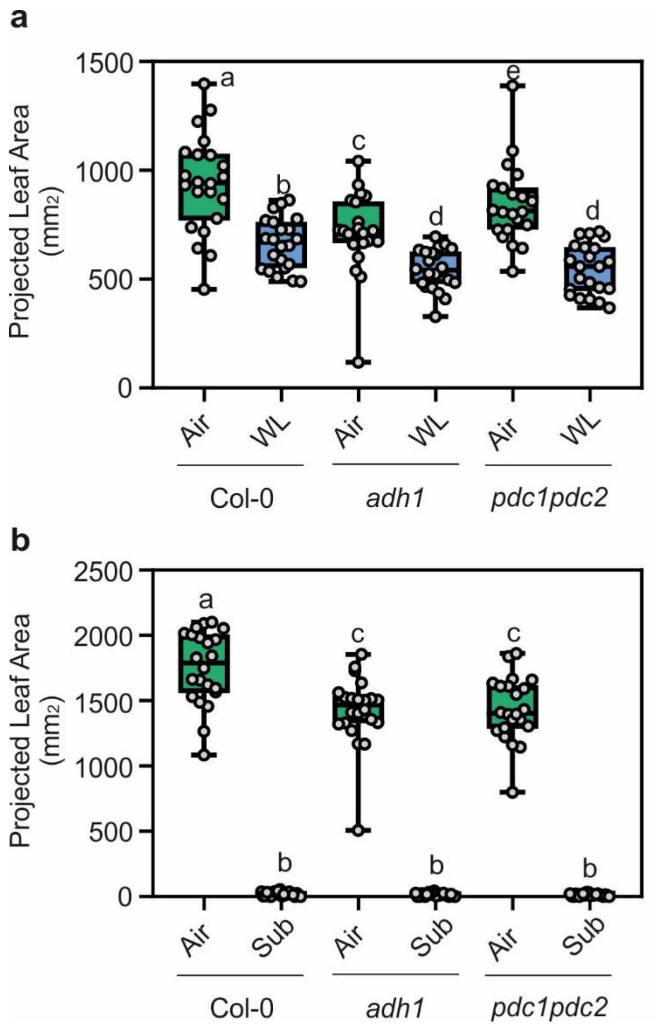

Figure S2. Effect of waterlogging and submergence on plant size in separate, independent experiments. **(a)** Plant size (Projected Leaf Area) in aerobic plants (green box) compared to plants that were waterlogged (blue box). Data were taken at day 28. **(b)** Plant size (Projected Leaf Area) in aerobic plants (green box) compared to plants that were submerged (blue box). Data were taken at day 33. The experimental set-up is the same as in Figures 4 and 6.

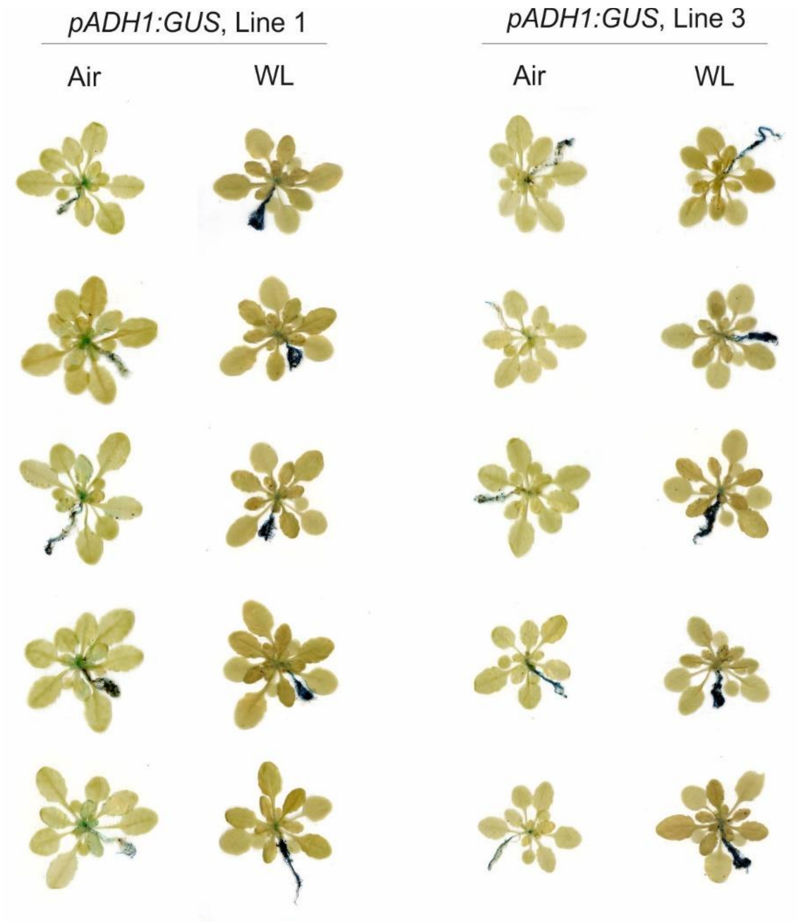

Figure S3. GUS staining of *pADH1:GUS* plants in air or 5-days waterlogging in wo independent transgenic lines. The experimental set-up is the same as in Figure 7a.

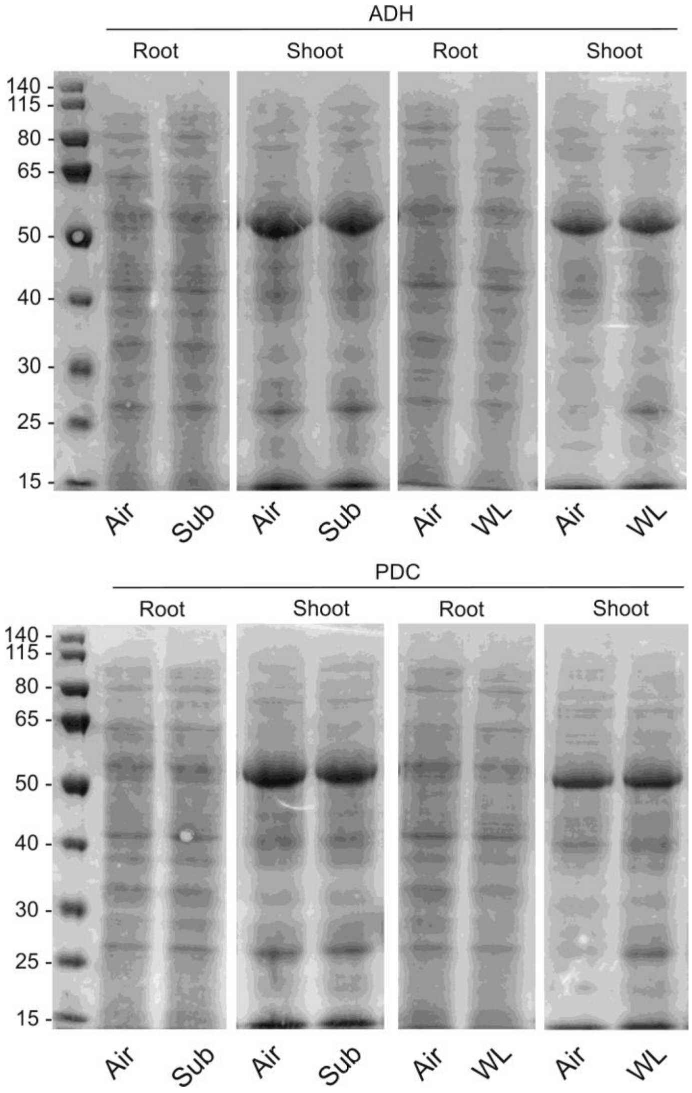

Figure S4. Gel staining with Amido Black demonstrating equal loading in the SDS-PAGE utilized for the western-blot shown in Figures 7 and 8.

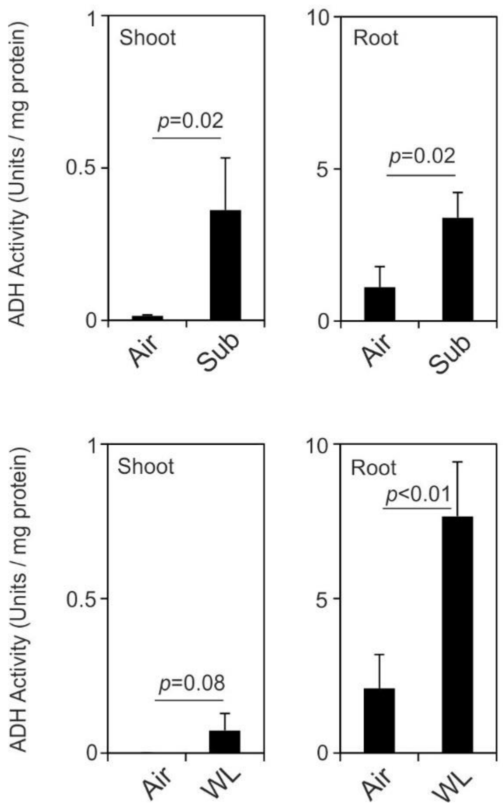

Figure S5. Activity of ADH in the samples utilized for the western-blot shown in Figures 7 and 8. One unit is the amount of enzyme leading to a change in A340 of 0.1 in one minute.

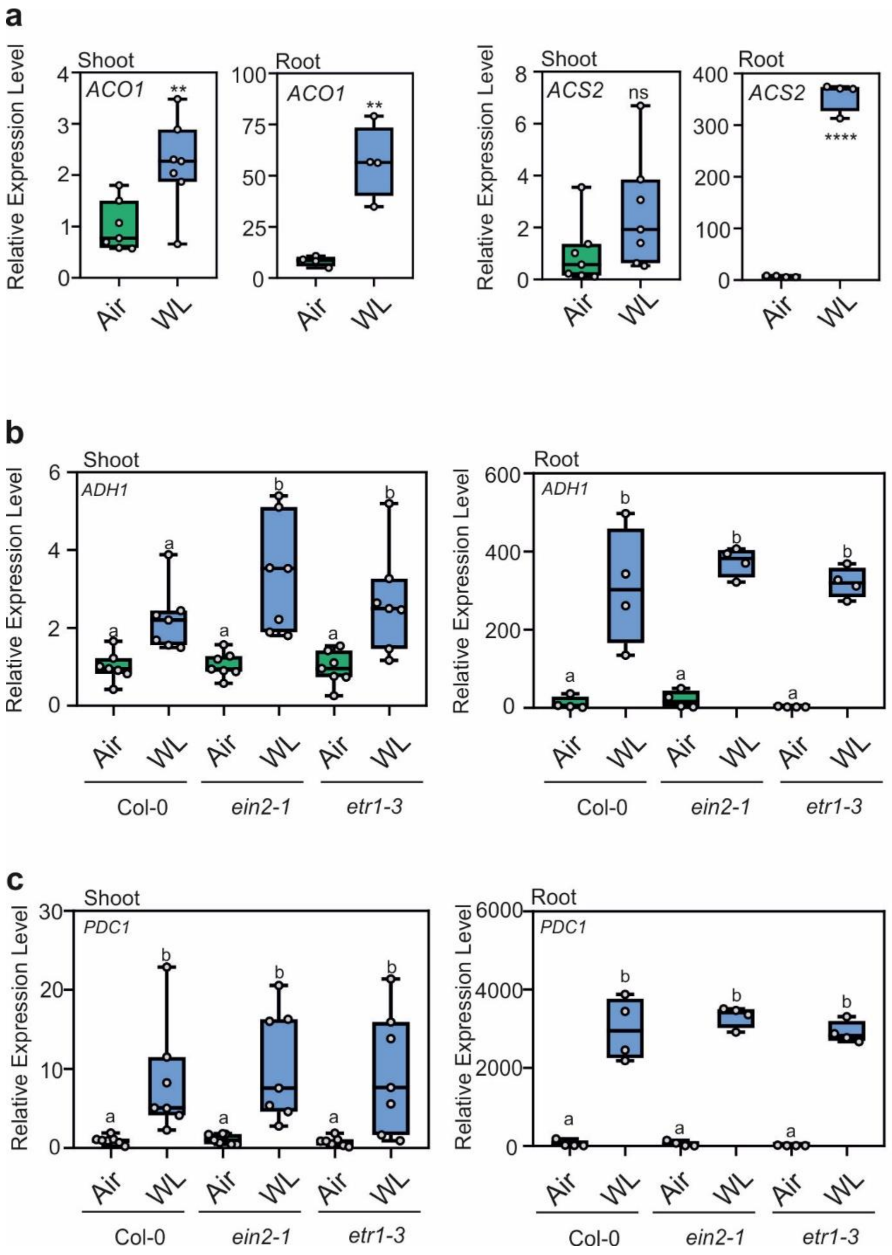

Figure S6. Role of ethylene in the induction of *ADH1* and *PDC1* in waterlogged plants. **(a)** Induction (RT-qPCR) of *ACO1* and *ACS2* by 5-days waterlogging; Statistically significant differences is indicated by the asterisk (T-test, pairwise comparison at each time point, *=p<0.05; **=p<0.01; ***=p<0.001; ****=p<0.0001). **(b)** Expression of *ADH1* in aerobic (air) and 5-days waterlogged (WL) plants. The genotypes used are shown in figure. Different letters indicate statistically significant differences (two-way ANOVA). **(c)** Expression of *PDC1* in aerobic (air) and waterlogged (WL) plants. The genotypes used are shown in figure. Different letters indicate statistically significant differences (ANOVA).

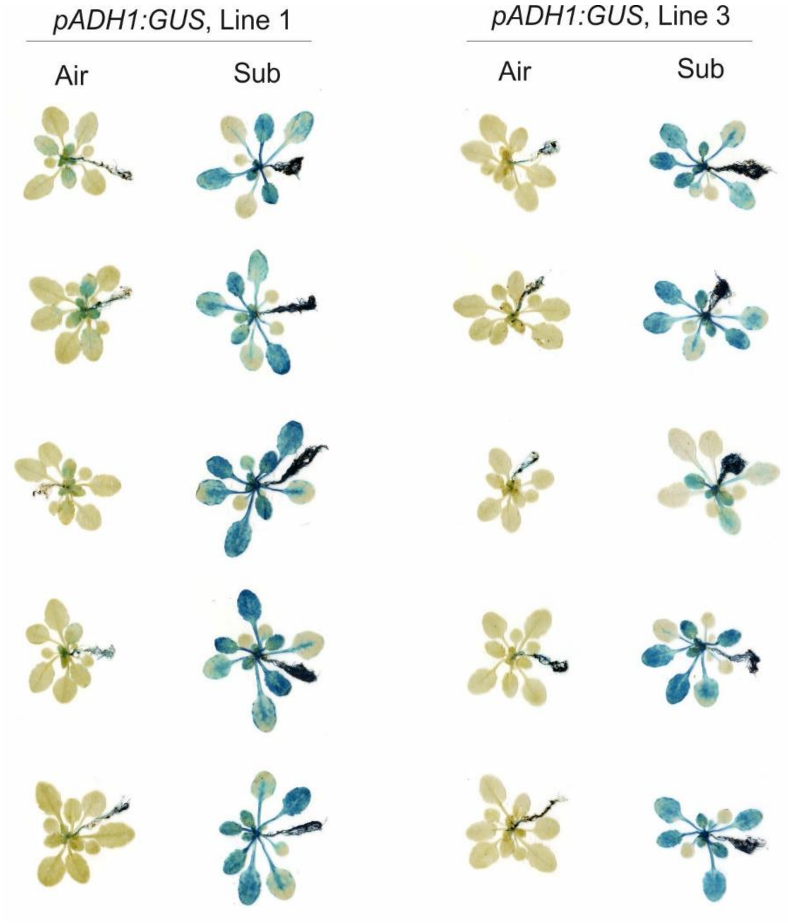

Figure S7. GUS staining of pADH1:GUS plants in air or 5-days waterlogging in wo independent transgenic lines. The experimental set-up is the same as in Figure 8a.

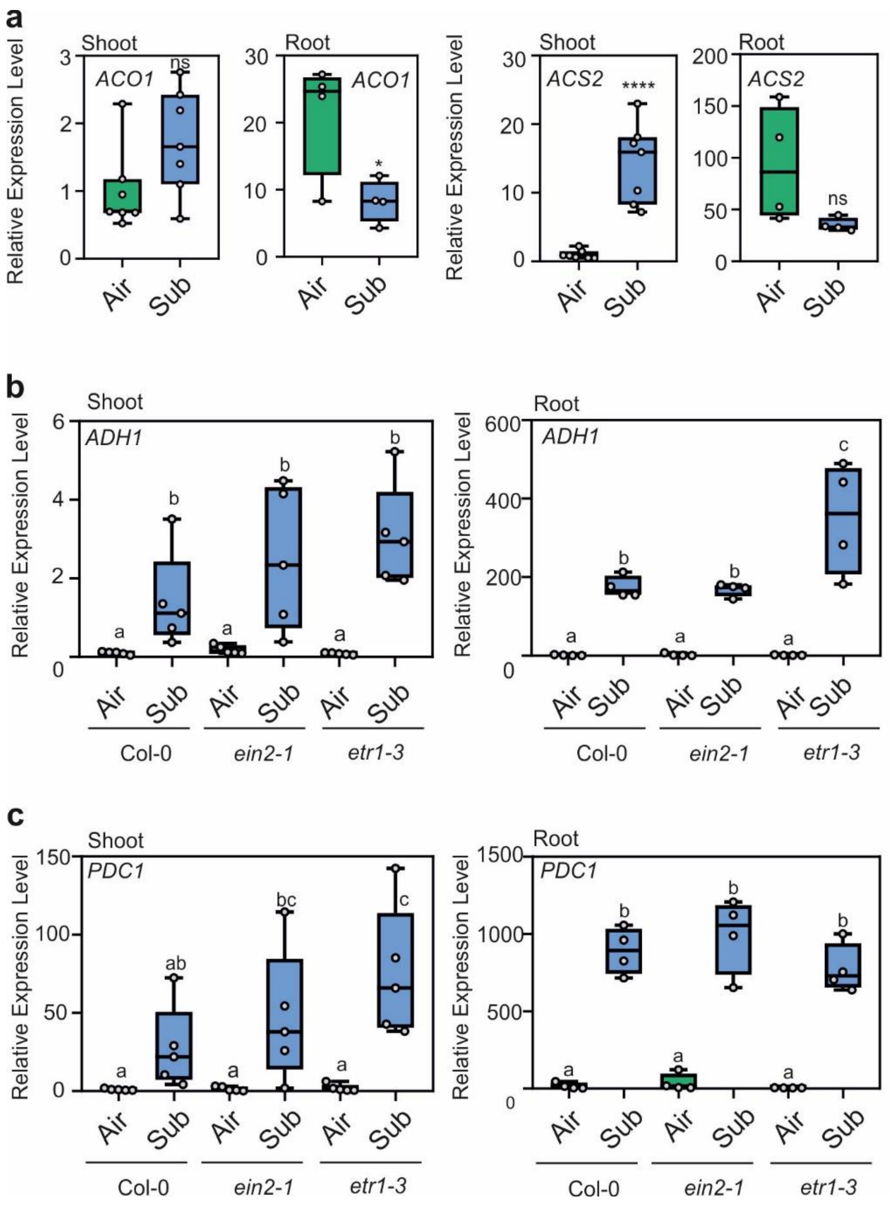

Figure S8. Role of ethylene in the induction of *ADH1* and *PDC1* in submerged plants. **(a)** Induction (RT-qPCR) of *ACO1* and *ACS2* by 35h submergence; Statistically significant differences is indicated by the asterisk (T-test, pairwise comparison at each time point, *=p<0.05; **=p<0.01; ***=p<0.001; ****=p<0.0001). **(b)** Expression of *ADH1* in aerobic (air) and 35h submerged (Sub) plants. The genotypes used are shown in figure. Different letters indicate statistically significant differences (two-way ANOVA). **(c)** Expression of *PDC1* in aerobic (air) and submerged (Sub) plants. The genotypes used are shown in figure. Different letter indicates statistically significant differences (ANOVA).

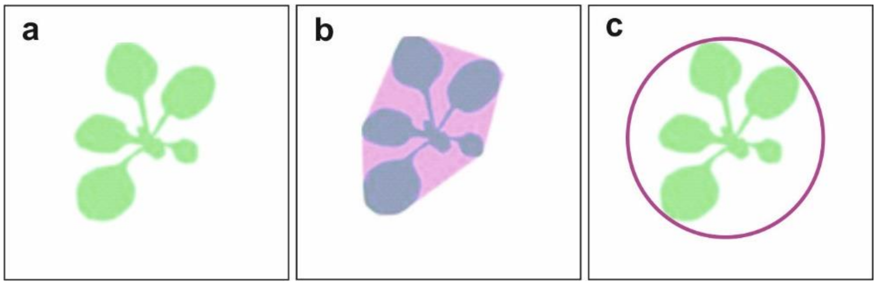

Figure S9. Description of the phenotypic parameters measured. (**a**) Projected Leaf Area: area of the rosette. (**b**) Compactness: Rosette Area/Conhull area. (**c**) Rosette Area / Area of min enclosing circle.
